# Osa-miR169 Negatively Regulates Rice Immunity against the Blast Fungus *Magnaporthe oryzae*

**DOI:** 10.3389/fpls.2017.00002

**Published:** 2017-01-17

**Authors:** Yan Li, Sheng-Li Zhao, Jin-Lu Li, Xiao-Hong Hu, He Wang, Xiao-Long Cao, Yong-Ju Xu, Zhi-Xue Zhao, Zhi-Yuan Xiao, Nan Yang, Jing Fan, Fu Huang, Wen-Ming Wang

**Affiliations:** ^1^Rice Research Institute and Key Lab for Major Crop Diseases, Sichuan Agricultural University at WenjiangChengdu, China; ^2^Collaborative Innovation Center for Hybrid Rice in Yangtze River Basin, Sichuan Agricultural University at WenjiangChengdu, China; ^3^College of Agronomy, Sichuan Agricultural University at WenjiangChengdu, China

**Keywords:** microRNA, miR169, nuclear factor Y-A, target mimicry, rice blast

## Abstract

miR169 is a conserved microRNA (miRNA) family involved in plant development and stress-induced responses. However, how miR169 functions in rice immunity remains unclear. Here, we show that miR169 acts as a negative regulator in rice immunity against the blast fungus *Magnaporthe oryzae* by repressing the expression of nuclear factor Y-A (NF-YA) genes. The accumulation of miR169 was significantly increased in a susceptible accession but slightly fluctuated in a resistant accession upon *M. oryzae* infection. Consistently, the transgenic lines overexpressing miR169a became hyper-susceptible to different *M. oryzae* strains associated with reduced expression of defense-related genes and lack of hydrogen peroxide accumulation at the infection site. Consequently, the expression of its target genes, the NF-YA family members, was down-regulated by the overexpression of miR169a at either transcriptional or translational level. On the contrary, overexpression of a target mimicry that acts as a sponge to trap miR169a led to enhanced resistance to *M. oryzae*. In addition, three of miR169’s target genes were also differentially up-regulated in the resistant accession upon *M. oryzae* infection. Taken together, our data indicate that miR169 negatively regulates rice immunity against *M. oryzae* by differentially repressing its target genes and provide the potential to engineer rice blast resistance via a miRNA.

## Introduction

Small RNAs are a type of short non-coding RNAs involved in regulation of gene expression either by chromatin methyl modification or by mRNA cleavage or/and translation inhibition (Baulcombe, 2004). Based on the difference of origin and function, small RNAs are classified into microRNAs (miRNAs) and small interfering RNAs (siRNAs). Both miRNAs and siRNAs are involved in the regulation of diverse biological processes, including growth, development and responses to biotic and abiotic stresses (Katiyar-Agarwal and Jin, 2010; Chen, 2012; Khraiwesh et al., 2012). Currently, more than 28000 miRNAs are listed in miRNA database^1^. Increasing evidence indicates that miRNAs are involved in fine-tuning plant immunity against pathogen invasion (Padmanabhan et al., 2009; Katiyar-Agarwal and Jin, 2010). The first identified resistance-related miRNA is miR393 in *Arabidopsis*, which can be induced by the pathogen-associated molecular pattern (PAMP) peptide flg22 and positively contributes to resistance against avirulent *Pseudomonas syringae* DC3000 by silencing auxin receptors to suppress auxin signaling (Navarro et al., 2006). miR160a was functionally characterized as positive regulator, whereas, miR398b and miR773 as negative regulators of plant PAMP-triggered immunity (PTI) in *Arabidopsis* (Li Y. et al., 2010). In addition, several miRNA families act as resistance regulators to direct the silence of nucleotide-binding leucine-rich-repeat (NB-LRR) type disease resistance (*R*) genes in plants (Baldrich and San Segundo, 2016). For example, nta-miR6019 and nta-miR6020 in tobacco guide the cleavage of transcripts of the Toll and Interleukin-1 receptor-NB-LRR immune receptor *N* that confers resistance to tobacco mosaic virus (TMV) (Li et al., 2012). In potato, three highly abundant miRNA families, miR1507, miR2109 and miR2118, target the conserved sequences in the transcripts of NB-LRRs, and trigger the production of trans-acting siRNAs, most of which are matched to over 60% of the NB-LRR-encoding genes in potato (Zhai et al., 2011). In tomato, miR482 targets the coiled-coil (CC)-NB-LRR-encoding genes and causes production of secondary siRNAs, which target other defense-related genes (Shivaprasad et al., 2012). In addition, miR482f and miR5300 also mediate silencing of the expression of resistance-related NB domain genes, but both the miRNAs are repressed in resistant cultivars during infection of the fungus *Fusarium oxysporum* f. sp. *lycopersici*, which causes vascular wilt disease in tomato (Ouyang et al., 2014). In rice, many miRNAs are also proposed to be involved in regulation of immunity against *Magnaporthe oryzae*, although the mechanism is largely unknown (Li et al., 2014, 2016).

Rice is the staple food for more than half of the world’s population (Liu et al., 2014). Rice blast caused by the fungal pathogen *M. oryzae* is one of the most devastating diseases threatening rice production worldwide. Understanding the mechanism of resistance to this disease can underpin disease control and many breakthroughs have been achieved in the past decades. On one hand, it is well-known that rice mounts two-layered innate immunity to defend against the invasion of *M*. *oryzae*. The first layer of innate immunity is activated upon recognition of PAMPs, such as chitin, by the cell-surface located pattern recognition receptors (PRRs), such as CEBiP, OsCERK1, LYP4 and LYP6, which is known as PTI (Kaku et al., 2006; Shimizu et al., 2010; Liu et al., 2012). This layer of immunity is suppressed by effector proteins of *M*. *oryzae*, some of which are recognized by R proteins, leading to the second layer of immunity, called effector-triggered immunity (ETI) (Liu et al., 2014). To date, more than 25 *R* genes have been functionally characterized and some of them are widely exploited in rice blast-resistant breeding and production^2^ (Liu et al., 2014; Ma et al., 2015). On the other hand, increasing reports indicate that miRNA signaling pathway is involved in rice immunity against *M. oryzae* and miRNAs can act as either positive or negative regulators to fine-tune PTI and ETI. For example, silencing *OsDCL1*, the key gene for miRNA biogenesis, results in enhancement of resistance to virulent rice blast strains due to the constitutive expression of 13 pathogenesis-related (*PR*) genes and two PTI-related genes *OsKS4* and *OsNAC4* (Zhang et al., 2015). Recently, nine new miRNAs were identified by deep sequencing of small RNA libraries derived from leaves treated with *M. oryzae* elicitors (Baldrich et al., 2015). In addition, the *M. oryzae* elicitor-responsive miRNA, Osa-miR7695, was identified as a positive regulator for rice resistance against *M. oryzae* by down-regulating the expression of *OsNramp6* (*Natural resistance-associated macrophage protein 6*) (Campo et al., 2013).

In a previous study, we identified more than 30 miRNAs that are differentially responsive to *M. oryzae* infection by comparing deep sequencing data of small RNA libraries from resistant and susceptible accessions (Li et al., 2014). Among these miRNAs, miR169 family members were differentially accumulated in the resistant accession IRBLkm-Ts and the susceptible accession Lijiang xin Tuan Heigu (LTH) upon *M*. *oryzae* infection (Li et al., 2014). While the accumulation of miR169a and miR169b/c were increased in both accessions upon *M. oryzae* infection, miR169f/g and miR169h/i/j/k/l/m were increased in LTH but decreased in IRBLkm-Ts. Therefore, it is intriguing to investigate the integrative role of miR169 because all the miR169 isoforms share high sequence identity and target to the same batch of genes encoding nuclear transcription factor Y (NF-Y) (Wu et al., 2009; Li Y. F. et al., 2010; Zhou et al., 2010).

Nuclear transcription factor Y is a family of transcription factors specifically binding the CCAAT-box in the promoters of eukaryotic genes via heterotrimer comprised of three subunits: NF-YA, NF-YB and NF-YC (Mantovani, 1999). In plants, each subunit of NF-Ys is respectively encoded by a group of genes. For example, there are 11 NF-YA, 11 NF-YB and seven NF-YC in rice and 10 NF-YA, 10 NF-YB, and 10 NF-YC in *Arabidopsis* (Petroni et al., 2012), resulting in the formation of a flexible and complicated transcription factor system that may regulate plant responses to different environmental conditions. In rice, eight of the 11 NF-YA genes are identified to be the authentic targets of miR169, including from NF-YA1 to NF-YA6, NF-YA10 and NF-YA11 (Wu et al., 2009; Li Y. F. et al., 2010; Zhou et al., 2010; Petroni et al., 2012). In fact, miR169 is a big microRNA family containing 17 known members representing nine different mature isoforms in rice (Zhao et al., 2007). Among all the isoforms, only miR169g is drought-induced, whereas, both miR169g and miR169n/o are inducible by high salinity, leading to down-regulation of the transcripts of *NF-YA2* (*Os03g29760*) (Zhao et al., 2007, 2009). In *Arabidopsis*, miR169/NF-YA module is linked with drought stress (Li et al., 2008), nitrogen (N) stress (Zhao et al., 2011; Liang et al., 2012), and associated with carbohydrate metabolism and cell expansion (Leyva-Gonzalez et al., 2012). In alfalfa, miR169/NF-YA module is involved in regulating symbiotic nitrogen fixation (SNF) process (Kaur et al., 2014) and freezing tolerance (Shu et al., 2016). In maize, the expression of Zma-miR169 and its target genes *ZmNF-YAs* is conversely responsive to drought, salt, and hormone stresses (Luan et al., 2014, 2015). In wheat, the expression of miR169 is reduced, whereas the expression of *NF-YAs*, are induced by N and P starvation (Qu et al., 2015). In rice, *OsNF-YA7* is induced by drought stress and the transgenic plants overexpressing *OsNF-YA7* exhibit ABA-independent tolerance to drought stress (Lee et al., 2015). These literatures indicate that miR169/NF-YA modules regulate tolerance to abiotic stresses in both monocots and dicots.

Recently, emerging evidence indicates that miR169/NF-YA modules also play roles in regulation of plant responses to biotic stresses. For example, in *Arabidopsis*, overexpression of miR169 abrogates the resistance phenotypes of *clv1* and *clv2*, the mutants of the LRR-receptor-like kinases *CLAVATA1* and *CLAVATA2*, to the bacterial wilt pathogen *Ralstonia solanacearum* via the suppression on NF-YA expressions (Hanemian et al., 2016). In rice, different miR169 isoforms are differentially accumulated in the resistant and susceptible accessions upon *M*. *oryzae* infection (Li et al., 2014, 2016), and are differentially responsive to elicitor treatment (Campo et al., 2013; Baldrich et al., 2015), indicating the involvement of miR169/NF-YA modules in rice responses to *M*. *oryzae*. However, it is unclear what roles the miR169/NF-YA regulation modules play in rice immunity against *M. oryzae*.

To figure out the integrative role of miR169 in rice immunity against the blast fungus, we first examined the abundance of different miR169 isoforms in the susceptible accession LTH and the resistant accession IRBL9-W upon *M. oryzae* infection. Then we constructed transgenic rice plants overexpressing miR169a and its target mimicry, respectively, and examined blast disease phenotypes of the transgenic lines. By examining the expression of miR169 target genes in transgenic lines and in susceptible/resistant accessions, we identified candidate *NF-YA* genes that might act as positive regulators for rice immunity. Taken together, our data demonstrate that miR169 acts as a negative regulator for rice immunity against *M. oryzae*.

## Materials and Methods

### Plant Materials and Growth Conditions

Rice (*Oryza sativa*) plants used in this study include the susceptible accession LTH, the resistant accession IRBL9-W, the *japonica* accession Taipei 309 (TP309) and its transgenic lines overexpressing miR169a, and the *indica* accession Kasalath and its transgenic lines overexpressing mimicry of miR169a (MIM169a). All rice plants were grown in a growth room maintained at 26°C and 70% relative humidity with a 14/10-h day/night regime. *Nicotiana benthamiana* plants were planted at 22°C with a 16/8 h light/dark photoperiod in a growth room and used for agro-infiltration experiments.

### Plasmid Construction and Genetic Transformation

To generate miR169a overexpressing transgenic plants, its genomic sequence containing 421 bp upstream and 500 bp downstream sequences was amplified from Nipponbare (NPB) DNA with primers *OsmiR169a-F* and *OsmiR169a-R* (Supplementary Table S2), and the amplified fragment was cloned into *Kpn*I-*Sal*I sites of the binary vector 35S-pCAMBIA1300, resulting in the overexpression construct p35S:miR169a. To make artificial target mimicry construct, we exploited the *Arabidopsis* gene *IPS1* (*INDUCED BY PHOSPHATE STARVATION1*) that contains sequences complement to miR399 with a mismatched loop at the expected miRNA cleavage site (Franco-Zorrilla et al., 2007). Artificial target mimicry sequences of miR169a were inserted into the *IPS1* to replace the miR399 target site with primers miR169-IPS1-F, miR169-IPS1-R, miR169mimic-F, and miR169mimic-R (Supplementary Table S2) as described previously (Franco-Zorrilla et al., 2007), and cloned into *Kpn*I-*Spe*I sites of binary vector 35S-pCAMBIA1300, resulting in the overexpression construct p35S:MIM169a. Construct p35S:miR169a was transformed into TP309 and construct p35S:MIM169a was transformed into Kasalath via *Agrobacterium tumefaciens* (strain GV3101)-mediated transformation and the transgenic plants were screened as previously described (Li et al., 2014). To express the YFP-tagged 3′-UTRs of target genes, the 3′-UTRs of *Os03g29760* (*NF-YA2*), *Os03g44540* (*NF-YA3*), and *Os03g48970* (*NF-YA4*) containing the target site of miR169 were amplified from Nipponbare (NPB) cDNAs using gene-specific primers (Supplementary Table S2). The isolated fragments were then fused to the C-terminus of *YFP* and inserted into *Kpn*I-*Spe*I sites of binary vector 35S-pCAMBIA1300. *Agrobacterium* strain GV3101 was used for agroinfection assay in *N. benthamiana* (Huang et al., 2014).

### Pathogen Infection and Microscopy Analysis

Three *M. oryzae* strains, Y34, Guy11 and eGFP-tagged Zhong8-10-14 (GZ8), were used in this study. *M. oryzae* strains were cultured in complete medium at 28°C with 12-h/12-h light/dark cycles for sporulation. After 2 weeks, spores were collected and the inoculum concentration was adjusted to 5 × 10^5^ spores mL^–1^ for spray inoculation on three-leaf-stage plants (Qu et al., 2006). T3 seedlings of miR169a transgenic plants were used for spray inoculation. Disease phenotypes on the leaf two were recorded at 5 days post inoculation (dpi). The blast disease lesions were examined and classified into six types (0–5 type; 0–2 type is classified as resistant phenotype, and 3–5 type is classified as susceptible phenotype) based on the sporulation rate on the lesions in infected rice leaves following a previous report (Bonman et al., 1986). In brief, 0 = no disease lesions observed; 1 = small pinpoint-like disease lesions between two small vascular bundles; 2 = lesions with diameter 0.5–1 mm and develop over the two small vascular bundles but do not reach the big vascular bundles; 3 = disease lesions with diameter about 1–3 mm and develop between the two big vascular bundles; 4 = disease lesions with diameter about 3–4 mm and develop over the two big vascular bundles; 5 = disease lesions with diameter over 4 mm and develop over the main vein. For evaluation of the transgenic lines expressing the target mimicry of miR169a, 8-week old T0 plants were inoculated following the drop inoculation method (Park et al., 2012), and the negative transgenic plants were used as control. In brief, leaves from 8-week-old plants were slightly wounded with a mouse ear punch, and 5 μL of spore suspension (5 × 10^5^ spores mL^–1^) was added to the wound. Lesions were measured at 5 dpi. Relative fungal mass was calculated using the DNA level of *M. oryzae Pot2* against the rice genomic ubiquitin DNA level by qPCR (Park et al., 2012). To observe the infection process of *M. oryzae*, the eGFP-tagged *M. oryzae* strain GZ8 was inoculated on 5-cm-long leaf sheaths as described (Kankanala et al., 2007). The inoculated epidermal layer was excised and analyzed by Laser Scanning Confocal Microscopy (Nikon A1) at 12 and 36 hpi, respectively.

For examining H_2_O_2_ accumulation in infected rice leaves, we followed the procedure published previously by Xiao et al. (2003). DAB and trypan blue were used to stain H_2_O_2_ and fungal structures, respectively. Images were acquired with a microscope (Zeiss imager A2).

### Quantitative Reverse Transcription PCR Assay

Three-leaf-stage plants were inoculated with *M. oryzae* by spraying spore suspensions at a concentration of 5 × 10^5^ spores mL^–1^, and samples were collected at 0, 6, 12, 24, and 48 hpi. TP309 is used as the control of miR169a transgenic lines. For examining the target gene expression in transgenic lines, negative transgenic lines were used as the control of MIM169a transgenic lines. Total RNA was extracted from collected samples using TRIzol reagent (Invitrogen) and was reverse transcribed to cDNA using the SuperScript first-strand synthesis system primed by oligo dT (Invitrogen). To test the expression of miRNAs, NCode miRNA first-strand cDNA module (Invitrogen) was selected to elongate and reverse transcribe miRNA following the manufacturer’s instructions. Quantitative RT-PCR was performed using isoform-specific primers (Supplementary Table S2) and SYBR Green mix (TaKaRa). snRNA U6 served as internal reference for the detection of miRNAs in quantitative RT-PCR. The rice *ubiquitin* (*UBQ*) gene was selected as an internal reference for data normalization. The data were determined by a one-way ANOVA followed by *post hoc* Tukey HSD analysis with significant differences (*P* < 0.01).

### *Agrobacterium*-Mediated Transient Expression Assay in *N. benthamiana*

*Agrobacterium* strain GV3101 harboring the respective expression constructs in the binary vector pCAMBIA1300 was incubated at 28°C overnight in LB media containing rifampin (50 μg/mL), kanamycin (50 μg/mL), and carbenicillin (50 μg/mL) at a 250 r/min shaking table. The bacteria were collected at 3000 rpm for 5 min and resuspended in an MMA buffer (10 mM MES, 10 mM MgCl_2_, 100 μM AS), respectively. The Agrobacteria harboring the expression constructs were infiltrated into leaves of *N. benthamiana* for transient expression assay. Leaves were examined between 36 and 80 hpi for image acquisition using a Nikon A1 Confocal Laser Scanning Microscope (Nikon Instruments, Inc., Chengdu, China) as previously described (Huang et al., 2014). Western blotting analyses were performed following a previous protocol (Chen et al., 2008). In brief, 15 micrograms of total protein were electrophoresed on 10% SDS-PAGE gel, and the protein blot was reacted with anti-GFP sera (BBI Life Science) to detect and determine YFP accumulation.

## Results

### Different miR169 Isoforms Are Differentially Accumulated in the Susceptible and Resistant Accessions

**FIGURE 1:**
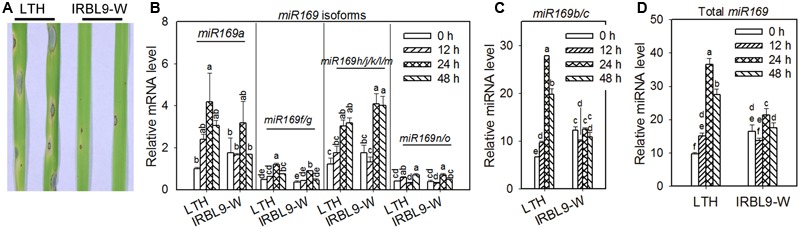
**Differential accumulation of *miR169* in susceptible and resistant accessions upon *Magnaporthe oryzae* infection. (A)** Representative leaf sections from the susceptible accession LTH and the resistant accession IRBL9-W show the blast disease phenotype. Three-leaf-stage seedlings were inoculated with Guy11 spore suspensions, and disease phenotypes were recorded at 5 dpi. Similar results were obtained in at least two independent experiments. **(B,C)** Differential accumulation of different miR169 isoforms in the indicated accessions upon *M. oryzae* infection. RNA was extracted at the indicated time points for qRT-PCR analysis. The transcriptional level of miR169 family members was normalized to miR169a in untreated LTH (0 h). SnRNA U6 served as an internal reference. **(D)** The sum accumulation of total miR169 in the indicated accessions upon *M. oryzae* infection. Values in **(B–D)** are means of three replications. Error bars indicate SD. The letters above the bars indicate significant differences (*P* < 0.01) as determined by a one-way ANOVA followed by *post hoc* Tukey HSD analysis. Similar results were obtained in at least two independent experiments.

To address the question of how different miR169 isoforms are differentially responsive to *M. oryzae* infection, we examined the accumulation of the five most abundant miR169 isoforms in the susceptible accession LTH and the monogenic resistant accession IRBL9-W that contains the resistance (R) gene *Pi-9* (Tsumematsu et al., 2000) and confers high resistance to *M. oryzae* (Khanna et al., 2015). LTH is widely used as a susceptible reference in blast inoculation experiments for it is susceptible to all *M. oryza*e strains ever tested (Lin et al., 2001). We first confirmed the disease phenotypes by inoculating the virulent strain Guy11 on three-leaf-stage seedlings. While large disease lesions were observed in LTH, small lesions were occasionally found in IRBL9-W (**Figure 1A**), indicating susceptibility and resistance, respectively. We next performed a time-course assay to examine the accumulation of miR169 isoforms in LTH and IRBL9-W by miRNA quantitative RT-PCR. According to the small RNA deep sequencing data in a previous report (Li et al., 2014), the accumulation of miR169a, miR169b/c, miR169f.1/g, miR169h/i/j/k/l/m, and miR169n/o is mostly abundant and thus was examined in this study, whereas, the accumulation of miR169d, miR169e, miR169p, and miR169q is rarely or not detected (below 50 reads or no reads per one million total reads) and thus not included in this study. Our data confirmed that the abundance of different miR169 isoforms was quite different, with the least abundant being for miR169f/g and miR169n/o, the most abundance being for miR169b/c, and the middle being for miR169a and miR169h/i/j/k/l/m (**Figures 1B,C**). Moreover, the accumulation pattern of miR169b/c was quite similar to the sum accumulation pattern of miR169 (**Figure 1D**). It is intriguing that the accumulation of all the tested miR169 isoforms was increased in LTH upon *M*. *oryzae* infection, whereas, the accumulation of miR169b/c was quite stable in IRBL9-W, although the accumulation of the other miR169 isoforms was also increased at certain time points (**Figures 1B,C**). However, the high abundance of miR169b/c might mask the fluctuation of the other isoforms so that its pattern is similar to the sum of miR169 (**Figure 1D**). Particularly, the total accumulation of all miR169 isoforms was highly up-regulated upon *M. oryzae* inoculation in LTH, but significantly down-regulated at 12 hpi, up-regulated at 24 hpi and back to the background level at 48 hpi in IRBL9-W (**Figure 1D**), which was consistent with the total reads in LTH and IRBLKm-Ts (in Supplementary Table S1 of Li et al., 2014). These data imply that miR169 may integratively act as a negative regulator in rice immunity against *M. oryzae*.

### Overexpressing miR169a Leads to Enhanced Susceptibility to *M. oryzae*

**FIGURE 2:**
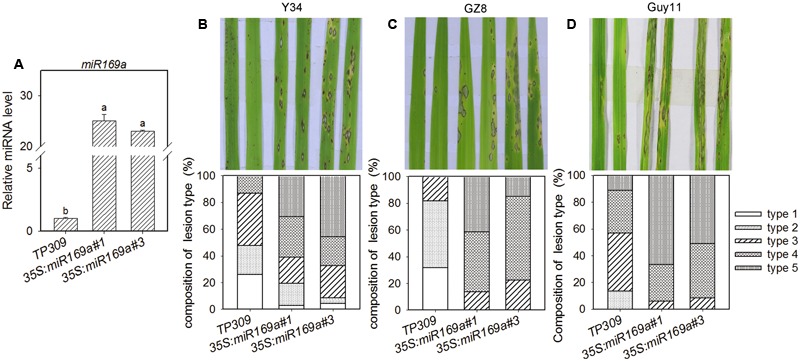
**Overexpressing miR169a results in enhanced susceptibility to *M. oryzae*. (A)** Comparison of miR169a accumulation in the transgenic lines harboring 35S:miR169a. SnRNA U6 served as the internal reference. The accumulation level of miR169a was normalized to that of the control plants. Values are means of three replications. Error bars indicate SD. The letters above the bars indicate significant differences (*P* < 0.01) as determined by a one-way ANOVA followed by *post hoc* Tukey HSD analysis. Similar results were obtained in at least two independent experiments. Comparison of disease phenotypes and composition of disease lesion types caused by the *M. oryzae* strain Y34 **(B)**, GZ8 **(C)** and Guy11 **(D)**, respectively. Lesions in 30 diseased leaves were analyzed at 5 dpi. Similar results were obtained in at least two independent experiments.

All miR169 isoforms target the same eight NF-YA genes (Wu et al., 2009; Li Y. F. et al., 2010; Zhou et al., 2010) because of their high sequence identity (Supplementary Figure S1). Thus, it is feasible to investigate the integrative role of all miR169 isoforms in rice immunity against *M. oryzae* by over expressing one of the miR169 isoforms, although the authentic miR169-NF-YA regulation module could be much more complicated due to different miR169 isoform has different expression pattern and is differentially responsive to different stimuli (Zhao et al., 2007, 2009). Following this scenario, we constructed rice transgenic lines overexpressing miR169a within the TP309 background. Two independent transgenic lines with high miR169a accumulation were identified and used for phenotypic analyses (**Figure 2A**). Three-leaf-stage seedlings were separately spray-inoculated with three *M. oryzae* strains with different virulence. While Y34 is an incompatible strain to TP309, Guy11 and the enhanced GFP-tagged Zhong8-10-14 (GZ8) are virulent strains. Disease phenotypes were recorded at 5 dpi. All the three strains formed more and larger disease lesions in the transgenic lines than those in the control plants (**Figures 2B–D**), indicating enhanced susceptibility by overexpression of miR169a. Then, the composition of lesion types was quantitatively analyzed. While the Y34-infected lesions in the control plants were mostly scored as types 1–2 (resistant phenotype), the lesions in the transgenic lines overexpressing miR169a were scored as types 3–5 (susceptible phenotype, **Figure 2B**), indicating that overexpression of miR169a converted the partial resistance into fully susceptibility to Y34. Moreover, the susceptible lesion types (3–5) of GZ8 and Guy11 in the control plants were all exacerbated in the transgenic lines overexpressing miR169a. The percentage of susceptible lesion types (types 3–4) of GZ8 was increased from 50% in the control plants to 80% in the transgenic lines (**Figure 2C**). Guy11-infected susceptible lesion types accounting for more than 80% in the control plants were exacerbated into 100% in the transgenic lines overexpressing miR169a, in which type 5 was increased from 10% in the control plants to more than 50% in the transgenic lines overexpressing miR169a (**Figure 2D**). These data demonstrate that overexpressing miR169a makes the susceptibility of TP309 to these strains into hyper-susceptibility.

### Overexpressing miR169a Results in Reduced Defense Responses

**FIGURE 3:**
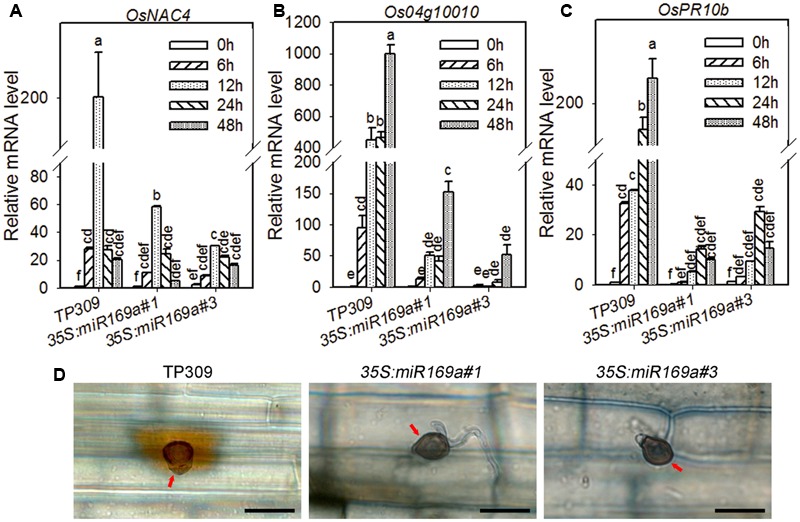
**Overexpressing miR169a results in compromised defense responses. (A–C)** Expression pattern of the indicated defense-related genes in the transgenic lines harboring 35S:miR169a and the control plants, respectively, upon *M. oryzae* infection. RNA was extracted at the indicated time points for qRT-PCR analysis. The indicated mRNA level was normalized to that in the untreated control plants (0 h). Values are means of three replications. Error bars indicate SD. The letters above the bars indicate significant differences (*P* < 0.01) as determined by a one-way ANOVA followed by *post hoc* Tukey HSD analysis. Similar results were obtained in at least two independent experiments. **(D)** Representative leaf sections from the indicated lines show H_2_O_2_ accumulation at 2 dpi, respectively. Note that there was no or trace amounts of H_2_O_2_ accumulation in the transgenic lines overexpressing miR169a, but high level of H_2_O_2_ was observed around the appressorium (arrow) in the leaf cell from the control plants. The fungal structure and accumulated H_2_O_2_ were stained by Trypan blue and DAB, respectively. Bars = 10 μm. Similar results were obtained in at least two independent experiments.

To explain why overexpression of miR169a leads to enhanced susceptibility, we examined typical defense responses, including expression levels of defense-related genes, production of hydrogen peroxide (H_2_O_2_) and microscopic pathogenesis of *M*. *oryzae*. First, three-leaf-stage seedlings were inoculated with *M. oryzae* strains Guy11. Then, the expression levels of three defense-related genes were examined by quantitative RT-PCR, including the two defense marker genes, *Oryza sativa pathogenesis-related protein10b* (*OsPR10b*) (Yamaguchi et al., 2013) and *Os04g10010* (Schwessinger et al., 2015), and *OsNAC4*, an earlier induced basal defense-related gene (Park et al., 2012). The three genes were significantly induced in the wild type at 6 hpi of *M. oryzae* (**Figures 3A–C**), in which *OsNAC4* was peaked at 12 hpi, whereas *OsPR10b* and *Os04g10010* were further increased along with the time points. On the contrary, the expression levels of these genes were much lower and the induced time was delayed in the transgenic lines, although their expressions were also induced (**Figures 3A–C**). Next, we examined the production of H_2_O_2_ upon inoculation of *M. oryzae*. Consistent with the reduced expression of defense-related genes, H_2_O_2_ was hardly detected in the infected sheath cells of transgenic lines at 24 hpi, while easily observed in the control plants at the same time point (**Figure 3D**). These data indicate that overexpression of miR169a compromises *M. oryzae*-related defense responses in rice plants.

**FIGURE 4:**
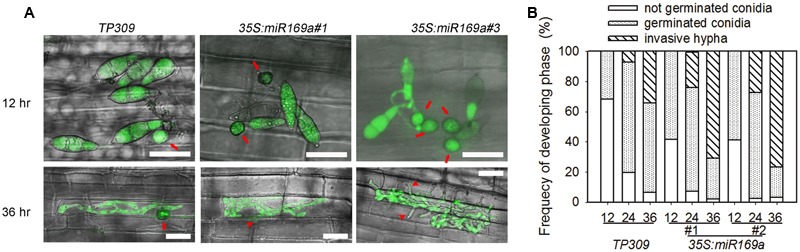
**Overexpressing miR169a leads to increased *M. oryzae* growth. (A)** Representative Laser Scanning Confocal Microscopy (LSCM) images show the growth of the *M. oryzae* strain GZ8 at 12 hours post inoculation (hpi) and 36 hpi on sheath cells of the indicated transgenic lines harboring 35S:miR169a and the control plants, respectively. Note that the appressoria (arrows) were formed at 12 hpi on miR169a-overexpression plants but delayed on control plants. Invasive hyphae were formed at 36 hpi on both control and miR169a-overexpressing plants, but only extended into the neighbor cells (arrowheads) of miR169a-overexpressing plants. Bars = 20 μm. **(B)** The quantitative analysis of *M. oryzae* growth. More than 200 conidia in each lines were analyzed. Similar results were obtained in at least two independent experiments.

In addition, we compared the infection process of *M. oryzae* strain GZ8 on leaf sheath of the wild type and the transgenic lines by using Laser Scanning Confocal Microscopy. At 12 hpi, more than half of the inoculated spores germinated and formed appressoria on the sheath from the transgenic lines overexpressing miR169a, which was in contrast to no or seldom appressoria formation on the sheath from the control plants (**Figures 4A,B**). At 36 hpi, more than 70% spores formed the invasive hyphae in the transgenic lines, which was in contrast to about 30% invasive hyphae formed in the control plants (**Figures 4A,B**). These results indicate that overexpressing miR169 can compromise rice defense responses so as to facilitate *M. oryzae* invasion at the early infection stage.

### miR169a Down-Regulates the Expression of Target Genes at Both Transcriptional and Translational Levels

**FIGURE 5:**
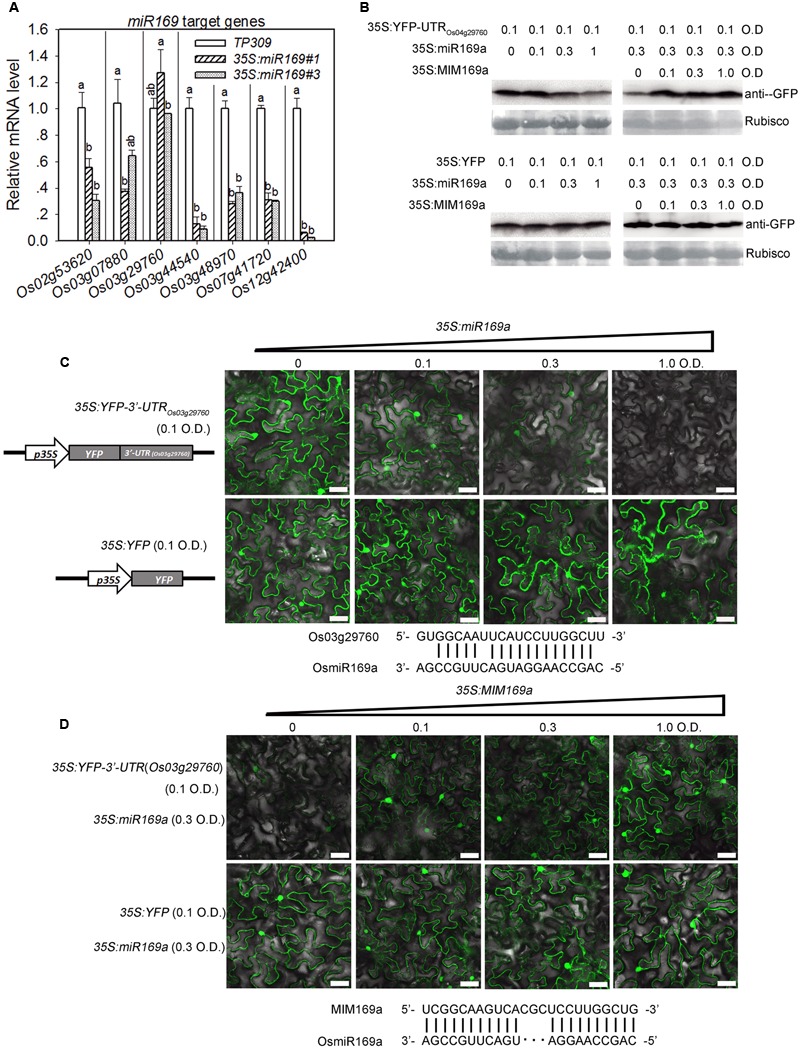
**MiR169a represses the expression of its target genes at transcriptional or translational level. (A)** Comparison of the relative mRNA levels of the indicated miR169’s target genes in the indicated lines. RNA was extracted in the indicated lines for qRT-PCR analysis. The mRNA level was normalized to that in the control plants. Values are means of three replications. Error bars indicate SD. The letters above the bars indicate significant differences (*P* < 0.01) as determined by a one-way ANOVA followed by *post hoc* Tukey HSD analysis. Western blotting analysis **(B)** and confocal images **(C,D)** show that miR169a suppressed the protein accumulation of *Os03g29760* but did not affect the protein level of YFP control. The indicated YFP-3′-UTR*_Os03g29760_* and YFP-based reporter constructs were transiently expressed alone or co-expressed with miR169a **(C)** or/and the miR169 target mimicry MIM169a **(D)** in *Nicotiana benthamiana* leaves using *Agrobacterium*-mediated infiltration at the indicated optical density (O.D.) concentration. Protein extracts from the same amount of infiltrated leaves were subjected to Western blot analysis using anti-GFP sera. The Coomassie Blue stained Rubisco indicated the equal sample loading **(B)**. The alignments of miR169a with *Os03g29760* target sequence **(C)** and MIM169a with miR169a **(D)** were listed below the images, respectively. Size bars, 40 μm. Similar results were obtained in at least two independent experiments.

All of the known target genes of miR169a encode NF-YA (Supplementary Table S1) (Wu et al., 2009; Li Y. F. et al., 2010; Zhou et al., 2010). As the role of miRNAs is to suppress target gene expression, we speculated that the expression of the target genes should be reduced in the lines overexpressing miR169a. To this end, we examined the mRNA levels of target genes in the lines overexpressing miR169a by quantitative RT-PCR. As anticipated, the transcription levels of six target genes, including *Os02g53620* (*NF-YA11*), *Os03g07880* (*NF-YA1*), *Os03g44540* (*NF-YA3*), *Os03g48970* (*NF-YA4*), *Os07g41720* (*NF-YA6*) and *Os12g42400* (*NF-YA10*), were repressed significantly in the transgenic lines; particularly, the expression of *Os03g44540* (*NF-YA3*) and *Os12g42400* (*NF-YA10*) was down-regulated to less than 20% of that in the control plants (**Figure 5A**). However, the transcription level of *Os03g29760* (*NF-YA2*) was significantly increased in one line and did not change in the other line (**Figure 5A**), and the simplest explanation is that miR169a may repress its translation or that *Os03g29760* (*NF-YA2*) is feedback regulated by its products. Then, we established an YFP-based reporter system to examine whether miR169a inhibits translation of *Os03g29760*. To this end, we made a construct expressing the Yellow Fluorescent protein (YFP) with the 3′-UTR of *Os03g29760* as the terminator (YFP-3′-UTR*_Os03g29760_*) because the target sites of miR169a on all of the eight NF-YA genes are located in the 3′-UTR (Supplementary Figure S2). Next, YFP-3′-UTR*_Os03g29760_* was separately expressed or co-expressed with miR169a in *N. benthamiana* and the protein levels were compared by both Western blotting analysis and examining the intensity of YFP. The results showed that when expressed alone, YFP-3′-UTR*_Os03g29760_* was highly accumulated (**Figure 5B**) and expressed in both the cytoplasm and the nucleus (**Figure 5C**). However, its expression level was obviously reduced when miR169a was co-expressed, particularly at higher OD value of the infiltrated Agrobacteria. By contrast, the co-expression of YFP that did not contain the 3′-UTR of *Os03g29760* and miR169a did not affect the protein accumulation of YFP (**Figures 5B,C**). To further confirm the translation inhibition by miR169 on 3′-UTR of *Os03g29760*, we constructed the target mimicry of miR169a, MIM169a, which could act as a sponge to trap miR169a because of the insertion of three nucleotides between positions 10 and 11, and as a result, the cleavage on the formed double strand RNA was inhibited because of the loop formation at the mismatched nucleotides (Franco-Zorrilla et al., 2007). When YFP-3′-UTR*_Os03g29760_*, miR169a and MIM169a were co-expressed, the protein accumulation of YFP-3′-UTR*_Os03g29760_* was obviously increased with the addition of MIM169a, indicating that the suppression on YFP-3′-UTR*_Os03g29760_* by miR169a was recovered by MIM169a (**Figures 5B,D**). These results suggest that miR169a may authentically repress the expression of *Os03g29760* at translational level.

To figure out whether miR169 suppresses the expression of the other target genes at protein levels, we also tested the expression of two target genes by using the reporter system. As anticipated, the YFP intensity of YFP-3′-UTR*_Os03g44540_* and YFP-3′-UTR*_Os03g48970_* were both reduced by co-expression of miR169a, whereas when MIM169a was co-expressed, the suppression on YFP-3′-UTR*_Os03g44540_* and YFP-3′-UTR*_Os03g48970_* by miR169a was recovered (Supplementary Figure S3). These results are consistent with the transcriptional level of the two target genes, and demonstrate that miR169a can indeed repress the expression of the two target genes at protein levels.

Unfortunately, we did not detect the expression of *Os07g06470* (*NF-YA5*) by qRT-PCR and failed to amplify this gene to make a transient expression construct. It is unclear whether and how the expression of *Os07g06470* (*NF-YA5*) is regulated by miR169a.

### Expressing Target Mimicry of miR169a Leads to Enhanced Resistance to *M. oryzae*

**FIGURE 6:**
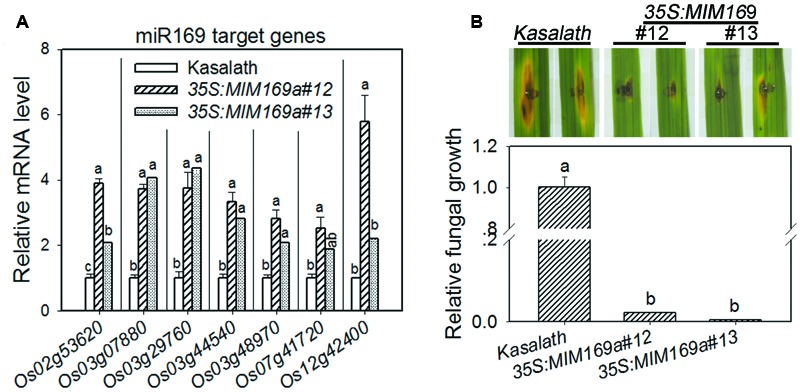
**Expression of the miR169a target mimicry results in enhanced resistance to *M. oryzae*. (A)** Comparison of the transcriptional level of miR169’s target genes in control plants and the transgenic lines expressing the miR169a target mimicry MIM169a. RNA was extracted in indicated lines for qRT-PCR analysis. mRNA level was normalized to that in control plants. Values are means of three replications. Error bars indicate SD. The letters above the bars indicate significant differences (*P* < 0.01) as determined by a one-way ANOVA followed by *post hoc* Tukey HSD analysis. **(B)** MIM169a transgenic lines display enhanced resistance to the *M. oryzae* strain GZ8. The relative fungal mass on the inoculated leaves of the indicated transgenic lines was measured by using the ratio of DNA level of *M. oryzae Pot2* against the rice genomic ubiquitin DNA level. Values are means of three replications. Error bars indicate SD. The letters above the bars indicate significant differences (*P* < 0.01) as determined by a one-way ANOVA followed by *post hoc* Tukey HSD analysis. Similar results were obtained in at least two independent experiments.

To further test the role of miR169a in rice immunity against the blast fungus, we constructed the target mimicry of miR169a, MIM169a, and obtained transgenic lines expressing MIM169a. Because overexpressing miR169a significantly repressed the expression of its target genes and led to enhanced susceptibility to *M. oryzae*, expressing MIM169a is predicted to up-regulate these target genes and result in enhanced resistance. Therefore, we first examined the expression of the target genes in the transgenic lines. As anticipated, the expression of all the detected target genes were significantly up-regulated in the two transgenic lines harboring the MIM169a construct (**Figure 6A**). Particularly, the expression of *Os12g42400* (*NF-YA10*) was up-regulated about sixfold in the transgenic line #12 and 2-fold in the transgenic line #13, whereas, the mRNA levels of all the other six target genes were generally up-regulated from 2- to 4-fold in the two independent MIM169a transgenic lines compared with that in the control plants. Next, we tested the resistance of the transgenic lines to *M. oryzae* strains GZ8. Indeed, the lesions on the leaves from transgenic lines were obviously smaller than that on the leaves from the control plants, and the fungal biomass on the transgenic lines were also decreased significantly than that on the control plants (**Figure 6B**), indicating that the up-regulation of miR169’s target genes alleviated rice susceptibility to *M. oryzae*. Taken together, these results confirmed that miR169 natively regulates rice immunity, whereas, its target genes, or some of its target genes, positively regulate rice immunity against *M. oryzae*.

### Different Target Genes of miR169 are Differentially Expressed in Susceptible and Resistant Rice Accessions

**FIGURE 7:**
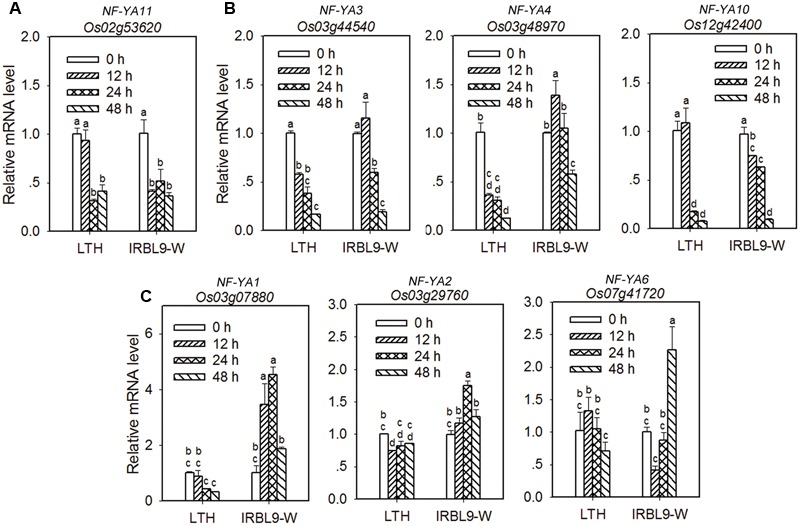
**Differential expression of miR169’s target genes in susceptible and resistant accessions upon *M. oryzae* infection. (A–C)** Expression of the indicated target genes in LTH and IRBL9-W upon *M. oryzae* infection. RNA was extracted at the indicated time points for quantitative RT-PCR analysis. mRNA level was normalized to that in untreated samples (0 h). Values are means of three replications. Error bars indicate SD. The letters above the bars indicate significant differences (*P* < 0.01) as determined by a one-way ANOVA followed by *post hoc* Tukey HSD analysis. Similar results were obtained in at least two independent experiments.

Now that all the detected seven target genes can be down-regulated by miR169 at either transcriptional or translational level, and up-regulated by the miR169’s target mimicry, it is questionable whether all of them similarly or equally regulate rice immunity against *M. oryzae*. To address this question, we performed a time course examination of their expression in LTH and IRBL9-W upon *M. oryzae* inoculation. According to the dynamic expression pattern, we divided the target genes into three groups. The first group contained one gene, *Os02g53620* (*NF-YA11*), its transcription level was decreased significantly upon *M. oryzae* infection in both LTH and IRBL9-W (**Figure 7A**). The second group included three genes, *Os03g44540* (*NF-YA3*), *Os03g48970* (*NF-YA4*), and *Os12g42400* (*NF-YA10*), their expression levels were decreased significantly and rapidly in LTH, but decreased slightly and slowly in IRBL9-W with *Os03g44540* (*NF-YA3*) and *Os03g48970* (*NF-YA4*) being transiently increased at 12 hpi and then decreased at the later time points of *M. oryzae* inoculation (**Figure 7B**). The third group included *Os03g07880* (*NF-YA1*), *Os03g29760* (*NF-YA2*) and *Os07g41720* (*NF-YA6*), their transcription levels were kept stable or decreased in LTH, but increased significantly at one or all examined time points in IRBL9-W (**Figure 7C**). Therefore, different target genes of miR169 are differently responsive to *M*. *oryzae* infection in the resistant and susceptible accessions and thus may act differently in rice immunity against *M*. *oryzae*. The three genes, *Os03g07880* (*NF-YA1*), *Os03g29760* (*NF-YA2*), and *Os07g41720* (*NF-YA6*) most likely contribute to the resistant phenotype of IRBL9-W.

## Discussion

The miR169 family is conserved and contains 17 members in rice (Zhao et al., 2007). Here, we provide data to show that miR169 acts as a negative regulator for rice immunity against the blast fungus *M*. *oryzae*. First, the sum accumulation of all miR169 isoforms was increased in the susceptible accession LTH, but fluctuated slightly in the resistant accession IRBL9-W (**Figure 1**), which was consistent with the previous high-throughput sequencing data in LTH and another resistant accession IRBLTs-Km (in Supplementary Table S1 of Li et al., 2014). Second, the transgenic lines overexpressing miR169a exhibited enhanced susceptibility to *M. oryzae* as indicated by the more severe disease phenotypes, down-regulated expression of defense-related genes, reduced defense response upon inoculation of *M. oryzae*, and accelerated *M. oryzae* invasion during the early infection period (**Figures 2**–**4**). Third, the transgenic plants expressing target mimicry of miR169a displayed enhanced resistance against *M. oryzae* (**Figures 6A,B**). In addition, the expression of all the tested seven NF-YA target genes were depressed at transcriptional or translational level by overexpressing miR169a, but up-regulated by the expression of a target mimicry of miR169a (**Figures 5** and **6**). Therefore, miR169 seems to negatively regulate rice immunity via multiple NF-YAs.

Nevertheless, each miR169 isoform may have its preferential target genes so that their expressions will be preferentially repressed when overexpressing a miR169 isoform. In *Arabidopsis*, overexpressing miR169d/e/f/g repressed the transcription of seven *AtNF-YA* genes, of which the three target genes, *AtNF-YA2, AtNF-YA8*, and *AtNF-YA10*, were down-regulated more significantly than the other four genes, including *AtNF-YA1, AtNF-YA3, AtNF-YA5*, and *AtNF-YA9* (Xu et al., 2014). On the contrary, expressing the target mimicry *MIM169defg* up-regulates the accumulation of *NF-YA2* and *NF-YA10* transcripts to levels significantly higher than that of other *NF-YA* target genes, suggesting that the miR169d/e/f/g isoform preferentially represses *NF-YA2* and *NF-YA10* (Sorin et al., 2014). In addition, co-expression of miR169 isoforms and *NF-YA5* in *N. benthamiana* revealed that miR169a was more efficient than miR169c in repressing *NF-YA5* at transcriptional level (Li et al., 2008). In the present study, overexpressing miR169a leads to repression of all the target genes tested. Particularly, the two target genes, *Os03g44540* (*NF-YA3*) and *Os12g42400* (*NF-YA10*), were repressed more than the other five *NF-YA* target genes (**Figure 5A**), indicating that miR169a may preferentially target *Os03g44540* (*NF-YA3*) and *Os12g42400* (*NF-YA10*).

In addition, different miR169 isoforms may differentially regulate their target genes through transcript cleavage or translation inhibition. The drought/salinity inducible miRNA isoforms, miR169g and miR169n/o, seems to preferentially mediate cleavage of *Os03g29760* (*NF-YA2*) and *Os07g41720* (*NF-YA6*) as indicated by the down-regulation of their transcripts when miR169g and miR169n/o were induced to higher levels (Zhao et al., 2009). In the present study, the transcription level of *Os03g29760* (*NF-YA2*) was not repressed in the transgenic lines overexpressing miR169a (**Figure 5A**). Potential reasons are that miR169a does not target *Os03g29760* (*NF-YA2*) or does not repress this target gene by transcript cleavage, or that the expression of *Os03g29760* (*NF-YA2*) is subjected to self-regulation to complement the down-regulation of the other *NF-YA* target genes. Therefore, we designed a transient expression reporter system to clarify whether miR169a could repress its target genes at protein level. Indeed, miR169a could repress the protein intensity of YFP-UTR*_Os03g29760_* when transiently co-expressed in *N. benthamiana*, implying that miR169a can regulate the expression of *Os03g29760* (*NF-YA2*) via translation inhibition. On the other hand, the expression of *Os03g29760* (*NF-YA2*) could also be suppressed by other miR169 isoforms via transcript cleavage, because its transcription was slightly decreased in the susceptible accession LTH upon *M. oryzae* infection, which was reversely correlated with the induced accumulation of miR169 isoforms (**Figure 1C**).

Finally, although miR169 targets the same batch of *NF-YA* genes, different target genes may function differently. Rice contains 11 *NF-YA* genes that are classified into several clades in a phylogenetic tree (Petroni et al., 2012), eight of the *NF-YA* genes are identified to be the target of miR169 (Wu et al., 2009; Li Y. F. et al., 2010; Zhou et al., 2010). *Os03g29760* (*NF-YA2*) and *Os07g41720* (*NF-YA6*) are rapidly down-regulated upon high salinity treatment, while the other *NF-YA* genes are not obviously changed (Zhao et al., 2009), indicating different *NF-YA* genes are differentially responsive to salt stress. In addition, different *NF-YA* genes are differentially induced by drought, salt and temperature stresses, but only *OsNF-YA7* (*Os08g09690*), a non-target of miR169, is highly induced by drought and salt treatment (Lee et al., 2015). In the present study, seven target *NF-YA* genes of miR169a were also differentially responsive to *M. oryzae* infection in LTH and IRBL9-W (**Figure 7A**). These target genes belong to different clades in a phylogenetic tree. While *Os02g53620* (*NF-YA11*) is not reported before, *Os03g07880* (*NF-YA1*), *Os03g29760* (*NF-YA2*), and *Os07g41720* (*NF-YA6*) are classified into class 1 clade, *Os03g48970* (*NF-YA4*) belongs to class 2, and *Os03g44540* (*NF-YA3*) and *Os12g42400* (*NF-YA10*) belong to class 3 clade (Petroni et al., 2012; Lee et al., 2015). It is interesting that the genes in the same clade displayed similar transcription patterns upon *M. oryzae* infection in LTH and IRBL9-W, suggesting that the *NF-YAs* in the different clade may play different roles in rice immunity against *M. oryzae*. For example, *Os02g53620* (*NF-YA11*) might act as a negative regulator in rice immunity against *M. oryzae* because its expression was rapidly and significantly decreased in the resistant accession IRBL9-W upon *M. oryzae* infection (**Figure 7A**), whereas, the three target genes, *Os03g07880* (*NF-YA1*), *Os03g29760* (*NF-YA2*) and *Os07g41720* (*NF-YA6*), might function as positive regulators because their expression levels were significantly up-regulated in the resistant accession IRBL9-W (**Figure 6C**). However, whether the three target genes, *Os03g48970* (*NF-YA4*), *Os03g44540* (*NF-YA3*), and *Os12g42400* (*NF-YA10*) function in rice immunity as positive or negative regulators requires further investigation because their expressions are differentially fluctuated in both the resistant and susceptible accessions upon *M. oryzae* infection. However, functional characterization of each of these genes via knockout approach could be difficult because of possible functional redundance among them. On the other hand, because all the seven target genes are significantly up-regulated in *MIM169a* transgenic lines which display enhanced resistance to *M. oryzae*, constructing transgenic lines overexpressing single target genes will be informative to understand the role of each target gene in regulation of rice immunity against *M. oryzae*. Taken together, our data demonstrated that miR169 acts as a negative regulator in rice immunity against *M. oryzae.* Overexpressing miR169a led to suppression of its target genes and enhanced susceptibility to *M. oryzae.* To the contrast, expressing the target mimicry of miR169a resulted in up-regulation of its target genes and enhanced resistance to *M. oryzae*. Therefore, it is possible to engineer rice blast resistance via managing the accumulation of miR169.

## Author Contributions

YL, S-LZ, J-LL, HW, X-LC, Y-JX, Z-XZ, Z-YX, and NY conducted the experiments. X-HH and FH conducted the field experiment. YL and JF supervised the study. YL wrote the manuscript. W-MW coordinated the overall study and edited the manuscript.

## Conflict of Interest Statement

The authors declare that the research was conducted in the absence of any commercial or financial relationships that could be construed as a potential conflict of interest.

## Footnotes

1 http://www.mirbase.org/index.shtml

2 http://www.ricedata.cn/gene/gene_pi.htm
