# Supplementary material for: Osa-miR169 Negatively Regulates Rice Immunity against the Blast Fungus Magnaporthe oryzae
Source: Front Plant Sci. 2017 Jan 17;8:2. doi: 10.3389/fpls.2017.00002 (PMC5239796; doi:10.3389/fpls.2017.00002)
Supplement: Supplementary file 1 [file Presentation_1.ZIP › Supplementary data/Supplementary Data.docx]

**Supplementary Data**

**Supplementary Figure S1. Alignment of the miR169 isoform sequences.**

**Supplementary Figure S2. Alignment of the miR169a with target sequences.**

**Supplementary Figure S3. MiR169a represses target genes at protein level.** Representative LSCM images show the subcellular localization pattern and protein accumulation of the indicated reporter constructs. The YFP-based reporter constructs were transiently co-expressed with miR169a or MIM169 constructs in *N. benthamiana* leaves with indicated concentration. Bars, 40 μm.

**Supplementary Table S1: List of miR169 target genes and their subclades.**

**Supplementary Table S2: List of primers used in this study.**
