# Supplementary material for: Osa-miR169 Negatively Regulates Rice Immunity against the Blast Fungus Magnaporthe oryzae
Source: Front Plant Sci. 2017 Jan 17;8:2. doi: 10.3389/fpls.2017.00002 (PMC5239796; doi:10.3389/fpls.2017.00002)
Supplement: Supplementary file 1 [file Presentation_1.ZIP › Supplementary data/Supplementary figures.pdf]

Figure S1.

**miR169a**	CAGCCAAGGATGACTTGCCGA
**miR169b/c**	CAGCCAAGGATGACTTGCCGG
**miR169f/g**	TAGCCAAGGATGACTTGCCTA
**miR169h/i/j/k/l/m**	TAGCCAAGGATGACTTGCCTG
**miR169n/o**	TAGCCAAGAATGACTTGCCTA

Figure.S2.

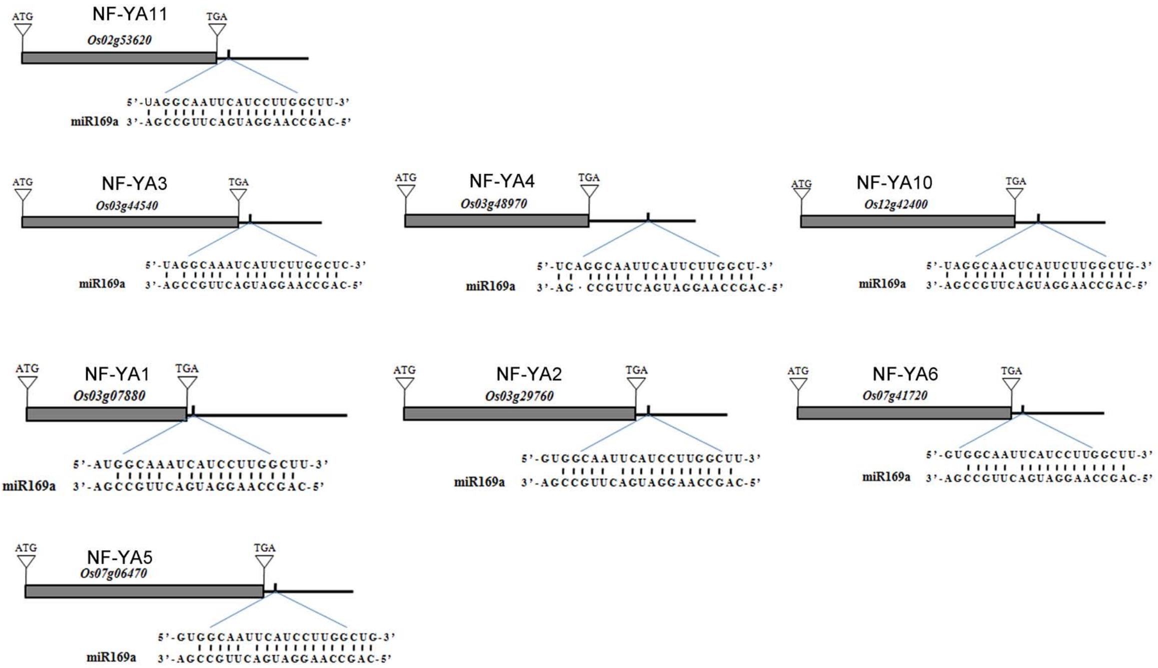
NF-YA11
Os02g53620
ATG
TGA
5'-UAGGCAAUUCAUCCUUGGCUU-3'
miR169a 3'-AGCCGUUCAGUAGGAACCGAC-5'
NF-YA3
Os03g44540
ATG
TGA
5'-UAGGCAAAUCAUUCUUGGCUC-3'
miR169a 3'-AGCCGUUCAGUAGGAACCGAC-5'
NF-YA4
Os03g48970
ATG
TGA
5'-UCAGGCAAUUCAUUCUUGGCU-3'
miR169a 3'-AG · CCGUUCAGUAGGAACCGAC-5'
NF-YA10
Os12g42400
ATG
TGA
5'-UAGGCAACUCAUUCUUGGCUG-3'
miR169a 3'-AGCCGUUCAGUAGGAACCGAC-5'
NF-YA1
Os03g07880
ATG
TGA
5'-AUGGCAAAUCAUCCUUGGCUU-3'
miR169a 3'-AGCCGUUCAGUAGGAACCGAC-5'
NF-YA2
Os03g29760
ATG
TGA
5'-GUGGCAAUUCAUCCUUGGCUU-3'
miR169a 3'-AGCCGUUCAGUAGGAACCGAC-5'
NF-YA6
Os07g41720
ATG
TGA
5'-GUGGCAAUUCAUCCUUGGCUU-3'
miR169a 3'-AGCCGUUCAGUAGGAACCGAC-5'
NF-YA5
Os07g06470
ATG
TGA
5'-GUGGCAAUUCAUCCUUGGCUG-3'
miR169a 3'-AGCCGUUCAGUAGGAACCGAC-5'

Figure S3.

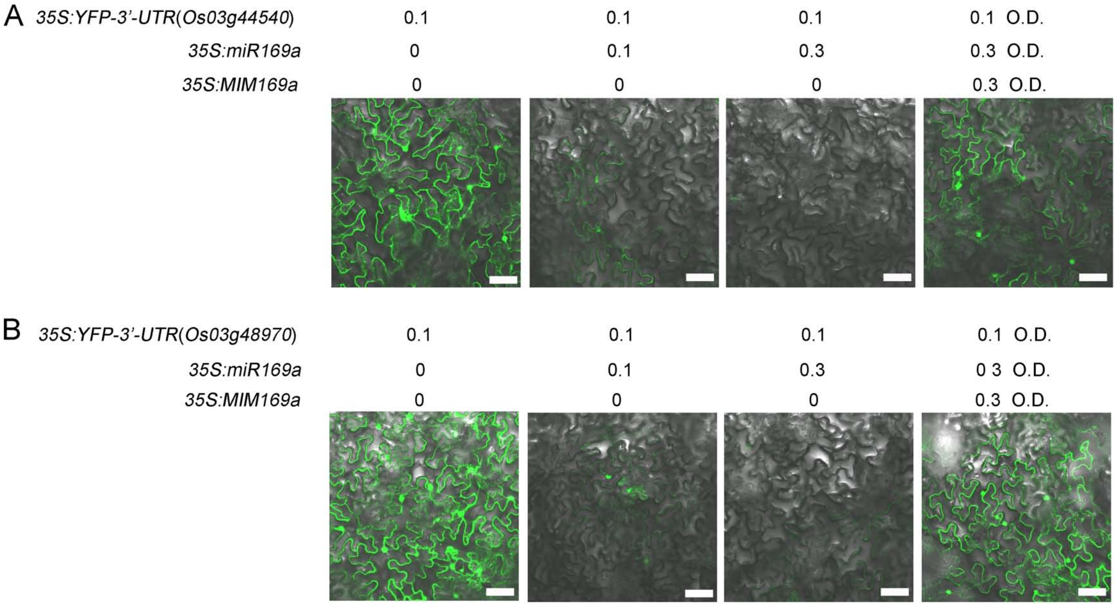
A
35S:YFP-3'-UTR(Os03g44540) 0.1 0.1 0.1 0.1 O.D.
35S:miR169a 0 0.1 0.3 0.3 O.D.
35S:MIM169a 0 0 0 0.3 O.D.
B
35S:YFP-3'-UTR(Os03g48970) 0.1 0.1 0.1 0.1 O.D.
35S:miR169a 0 0.1 0.3 0 3 O.D.
35S:MIM169a 0 0 0 0.3 O.D.
